# Societal importance of Antarctic negative feedbacks on climate change: blue carbon gains from sea ice, ice shelf and glacier losses

**DOI:** 10.1007/s00114-021-01748-8

**Published:** 2021-09-07

**Authors:** D. K. A. Barnes, C. J. Sands, M. L. Paulsen, B. Moreno, C. Moreau, C. Held, R. Downey, N. Bax, J. S. Stark, N. Zwerschke

**Affiliations:** 1 British Antarctic Survey, NERC, Cambridge, UK; 2 Australian Antarctic Division, Hobart, Australia; 3 Aarhus Universitet, Aarhus, Denmark; 4 Universidad Científica del Sur, Lima, Peru; 5 Université Libre de Bruxelles, Brussels, Belgium; 6 Alfred Wegner Institute, Bremerhaven, Germany; 7 Australian National University, Canberra, Australia; 8 South Atlantic Environmental Research Institute, Stanley, South Atlantic Falkland Islands

**Keywords:** Blue carbon, Ecosystem services, Sea ice, Nature-based solutions, Southern Ocean

## Abstract

Diminishing prospects for environmental preservation under climate change are intensifying efforts to boost capture, storage and sequestration (long-term burial) of carbon. However, as Earth’s biological carbon sinks also shrink, remediation has become a key part of the narrative for terrestrial ecosystems. In contrast, blue carbon on polar continental shelves have stronger pathways to sequestration and have increased with climate-forced marine ice losses—becoming the largest known natural negative feedback on climate change. Here we explore the size and complex dynamics of blue carbon gains with spatiotemporal changes in sea ice (60–100 MtCyear^−1^), ice shelves (4–40 MtCyear^−1^ = giant iceberg generation) and glacier retreat (< 1 MtCyear^−1^). Estimates suggest that, amongst these, reduced duration of seasonal sea ice is most important. Decreasing sea ice extent drives longer (not necessarily larger biomass) smaller cell-sized phytoplankton blooms, increasing growth of many primary consumers and benthic carbon storage—where sequestration chances are maximal. However, sea ice losses also create positive feedbacks in shallow waters through increased iceberg movement and scouring of benthos. Unlike loss of sea ice, which enhances existing sinks, ice shelf losses generate brand new carbon sinks both where giant icebergs were, and in their wake. These also generate small positive feedbacks from scouring, minimised by repeat scouring at biodiversity hotspots. Blue carbon change from glacier retreat has been least well quantified, and although emerging fjords are small areas, they have high storage-sequestration conversion efficiencies, whilst blue carbon in polar waters faces many diverse and complex stressors. The identity of these are known (e.g. fishing, warming, ocean acidification, non-indigenous species and plastic pollution) but not their magnitude of impact. In order to mediate multiple stressors, research should focus on wider verification of blue carbon gains, projecting future change, and the broader environmental and economic benefits to safeguard blue carbon ecosystems through law.

## Introduction

**Fig. 1:**
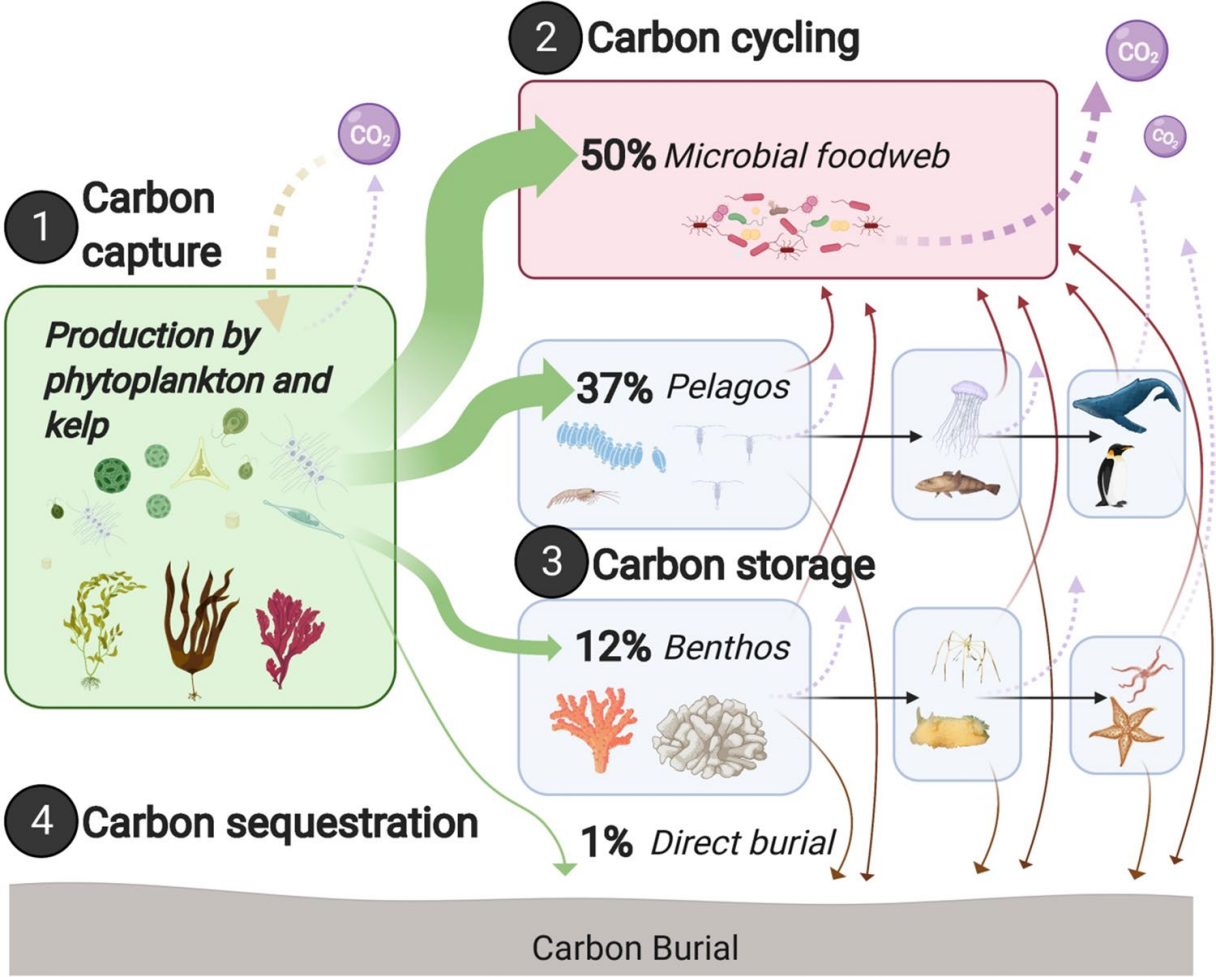
Initial fate of net carbon captured in primary production (green arrows) (see Henley et al. 2020). Blue carbon storage in pelagic and benthic primary consumers (37 + 12 = 49%) has similar fates; mainly recycling by microbes (red arrows) and by respiration (purple dashed arrows), some eaten by predators (black arrows) and 0–20% sequestration (brown arrows)

Halting biodiversity loss, mitigating climate change and improving societal quality of life are not mutually exclusive, and nature-based solutions (NbS) are an important part of achieving these aims simultaneously. Targeted nature protection, restoration and rewilding inherently starts with plants as the base of food webs, thus involving carbon capture (through photosynthesis) and storage (in the body of organisms). However, upon the death of these organisms, their bodies will pass through the food web and most of the carbon stored in them will ultimately be released as CO_2_ back into the water and atmosphere. So, the area/biomass of organisms capturing and storing carbon needs to be increased to remove more carbon than is being returned to the atmosphere to cause a net removal of CO_2_ from the atmosphere and thus mitigate climate change. Carbon capture on land is happening at increasing rates coincident with rising atmospheric CO_2_ levels (e.g., global greening, see Saban et al. 2018) and this is also happening in the sea with warming (phytoplankton blooms, see Arrigo et al. 2008). The fate of new carbon capture (primary production) has important implications to mitigating the impacts of climate change. Coastal wetlands (mangrove, sea grass and salt marshes) are amongst the most efficient at converting carbon capture into sequestration (total removal of carbon from the cycle for > 100 years), but they occupy < 1% of Earth’s surface and are all declining in size, despite strong restoration efforts (Duarte et al. 2005). The IPCC (2019) estimates that restoration of such habitats may sequester 0.20–0.84 GtCO_2_e a^−1^ (gigatonnes of carbon dioxide equivalent per year). Currently, blue carbon (carbon in marine organisms, Fig. 1) accounts for half the carbon buried in oceans (Duarte et al. 2005). Macroalgae can export 80% of production through their blades that can be shredded in storms and these fragments ultimately accumulate on the seabed (Krumhansl and Scheibling 2012). As much as 11% of the carbon from macroalgae may be sequestered in continental slope and deep abyss muds (Krause-Jensen and Duarte 2016). In contrast, only a small proportion of phytoplankton may be directly sequestered (Fig. 1) but total phytoplankton biomass is many orders of magnitude larger than macroalgae. Carbon from this short-lived primary production is stored by consumers in the food web, including benthos (Barnes 2015; Henley et al. 2020; Rossi and Rizzo 2020). On a global scale, blue carbon in polar seas is least considered, partly because the habitat types typically associated with carbon capture and sequestration (mangroves, salt marshes and seagrasses) do not occur there. Polar coasts and shallow seas have a very high potential for blue carbon storage and sequestration because of their rich and dense biota, large areas that will likely be ice-free in the future and high biomass pelagic communities with macroalgal kelp forests. Simultaneously, phytoplankton (microalgae) blooms in polar regions support massive populations of copepods, krill, higher predators (including birds, seals, toothed and baleen whales) and thousands of native and endemic benthos species (Trivelpiece et al. 2011; Rogers et al. 2020).

**Fig. 2:**
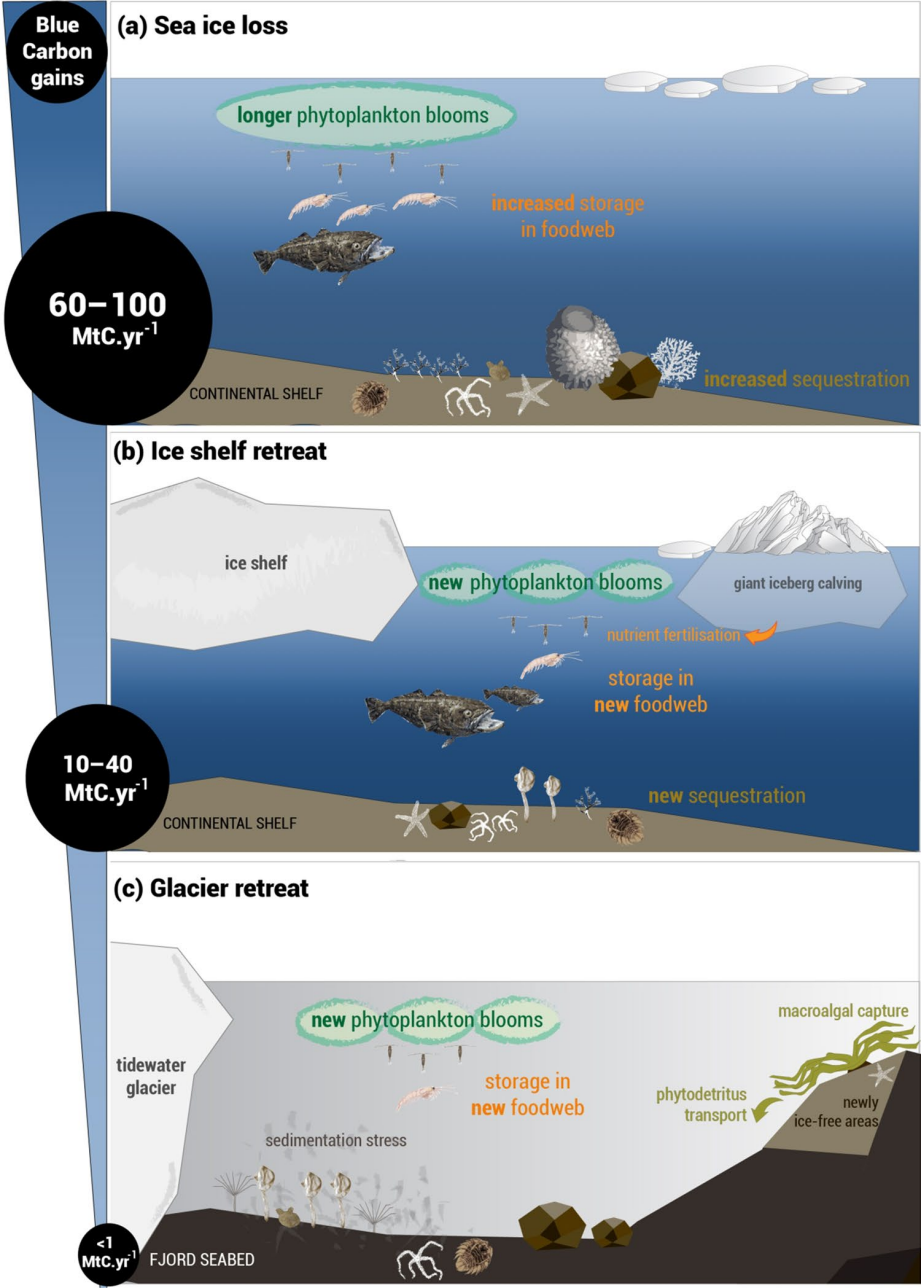
Main blue carbon changes with marine ice loss type on polar continental shelves. Increased carbon sequestration values are from Barnes et al. (2018, 2020). Although blue carbon gains are highlighted, marine ice losses can also have associated negative impacts from increased ice scour, albedo change, habitat loss and reduced ice shelf buttressing

The Arctic and Antarctic are warming and losing ice mass, but both warming and ice loss patterns are extremely complex in time and space, even within each polar region (see e.g., Turner and Comiso 2017). Marine ice loss allows increased levels of light and energy to enter the water and can change phytoplankton bloom duration, timing (Arrigo et al. 2008) and composition (Rogers et al. 2020). Thus, marine ice losses, caused by greenhouse gas–induced warming, can increase primary production (carbon capture or drawdown), which provides more food for longer periods of time for marine animals to produce more biomass (carbon storage, see Barnes 2015; Pineda Metz et al. 2020). However, it is also possible for ice loss to decrease regional productivity both in the Arctic and Antarctic (e.g. Wassmann and Reigstad 2011), due to potential future changes in stratification, which could reduce the amount of food that gets to the seabed. Polar continental shelves can be wide (1000 km in places), deep (1000 m in places) and muddy, so if polar blue carbon increased in these areas, it has high burial and sequestration prospects (Peck et al. 2010; Barnes and Sands 2017). Blue carbon on coastal polar continental shelves has been shown to increase in power (Mt C storage) with increased climate change (marine ice loss), thus effectively dampening it and working as a negative (mitigating) feedback loop on climate change (Barnes 2015). Despite being globally small carbon sinks (turnover and storage biomass), polar continental shelves nevertheless rank as three of the biggest four negative feedbacks on climate (Barnes et al. 2018). If these new emerging and increasing polar carbon sinks sustain their performance and are protected, they have great societal value (Gogarty et al. 2020; Bax et al. 2021). There are considerable uncertainties about, and growing threats to, the future efficiency and function of these cold-water carbon sinks. These include fishing (Thrush and Dayton 2002), ocean acidification (Orr et al. 2005), pollution (Waller et al. 2019), non-indigenous species (Hughes et al. 2020), warming (Ashton et al. 2017), ice scour and sedimentation in the shallows (Sahade et al. 2015) and the interactions of all of these (Gutt et al. 2015; Rogers et al. 2020). The current work attempts to outline the magnitude, processes, and future of blue carbon in relation to three differing but key marine ice loss types: seasonal sea ice, ice shelves and glaciers (Fig. 2).

## Seasonal sea ice

The ‘sea ice’ (component of marine ice) forms seasonally mainly by the autumn/winter freezing of the sea surface and its breakup in spring/summer. Where this frozen surface attaches to the shore, it is referred to as ‘fast ice’, but sea ice also includes icebergs and loose pieces ‘brash ice’ calved from glacier termini and ice shelf collapses. Of the three forms of marine ice, sea ice occupies by far the most area—2.5 to 20 million km^2^ in the Southern Ocean and ~ 4 to 16 million km^2^ in the Arctic. The Arctic sea ice maximum and Antarctic minimum is in March and the Arctic sea ice minimum and Antarctic maximum is in September, but all have decreased considerably in the last four decades (Turner and Comiso 2017). Turner and Comiso (2017) detail how the extensive and sustained sea ice losses around much of the Arctic and West Antarctica have contrasted with a long-term rise around East Antarctica until 2014 followed by catastrophic recent losses (there has since been a moderate recovery to 2021). In contrast, the pattern of change in sea ice in East Antarctica between 1979 and 2010 shows mixed signals on regional to local scales, with areas of strongly positive and negative trends occurring in relative proximity in some regions, e.g. Prydz Bay (Massom et al. 2013). The area of seabed overlain by sea ice is an order of magnitude larger than that of ice shelf (which in turn is [at least 3] orders of magnitudes larger than marine glacier area). We discuss the impacts of sea ice losses first as they dwarf those of ice shelf disintegration (> 1 million km^2^ vs 25,000 km^2^ respectively—see https://nsidc.org/).

When present, sea ice stabilises the water column, reduces gas and heat flux, limits light penetration and provides a novel and crucial habitat for many species including krill (Thomas 2005; Rogers et al. 2020). Patterns of change and variability in sea ice are influenced by the different elements of the marine ‘icescape’, including fast ice, polynyas and the marginal ice zone (Massom et al. 2013). Near the coast, sea ice immobilises icebergs minimising their drift and thus reduces their scouring of the seabed, leading to a significant correlation between sea ice duration, seabed scouring rate (Smale et al. 2008) and biodiversity dynamics (Gutt 2001). In reverse of this, the presence of large icebergs and glacier tongues can stabilise and anchor fast ice to coasts (Massom et al. 2013). Over the wider polar marine environment, there is also a strong relationship between the duration and timing of sea ice, phytoplankton blooms and secondary production (Barnes and Clarke 1994; Arrigo et al. 2008; Rogers et al. 2020). How sea ice indirectly drives zoobenthic carbon storage across different sea ice scenarios has been quantified in several different ways: (1) ocean scale; sample one taxon’s standing stock and annual increment across different years and seas and correlating with Earth Observation (remotely sensed) phytoplankton and sea ice conditions, then scale up from one to all taxa (Bryozoans in Barnes 2015). (2) Sea (intermediate) scale; sample standing stock of multiple taxa across multiple years in a sea with changing sea ice and phytoplankton performance (Pineda Metz et al. 2020, Souster et al. 2020). (3) Small scale; directly observe local sea ice, ice scour and measure primary and secondary production with high detail across multiple years (Barnes 2017).

There is considerable literature on the nature and magnitude of zoobenthic biomass around Arctic and Antarctic shelf seabeds (e.g. Arntz et al. 1994) and recently a subset of this has focused on quantifying the carbon storage and sequestration potential (Barnes et al. 2018; Pineda Metz et al. 2020; Souster et al. 2020). The geographic and bathymetric location of this (zoobenthic) carbon storage makes it logistically difficult, time-consuming and expensive to sample; hence, data are sparser than those in other seas. Each of the different approaches to help handle the extrapolation to continental scales to date have strengths and weaknesses, make many assumptions and have considerable error. Not least amongst these is that much polar sea ice overlays continental slope and deep (abyssal) seabed for which there has been little quantitative exploration of sea ice-production-carbon pathway change. Climate models cannot recreate recent sea ice extent change (to test how robust their future projections are), so to find linkages between these and potential biological responses, which itself are intensely patchy (at multiple scales) and complex, is lacking at the scales needed.

**Fig. 3:**
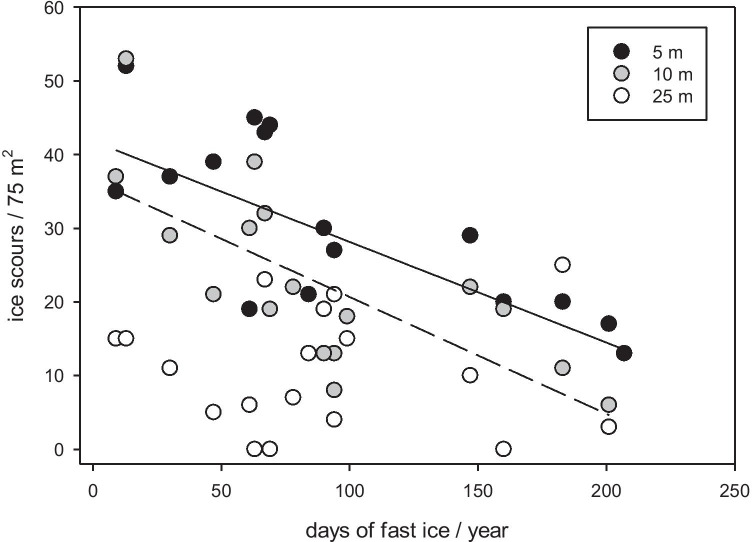
Duration of seasonal sea ice (fast ice) with iceberg scour frequency and depth at Ryder Bay, West Antarctic Peninsula. Continuous line is sea ice duration-ice scour relationship at 5-m depth and broken line is at 10-m depth (depth separated data from Barnes et al. 2018). No relationship is evident in deeper (25 m) water

**Fig. 4:**
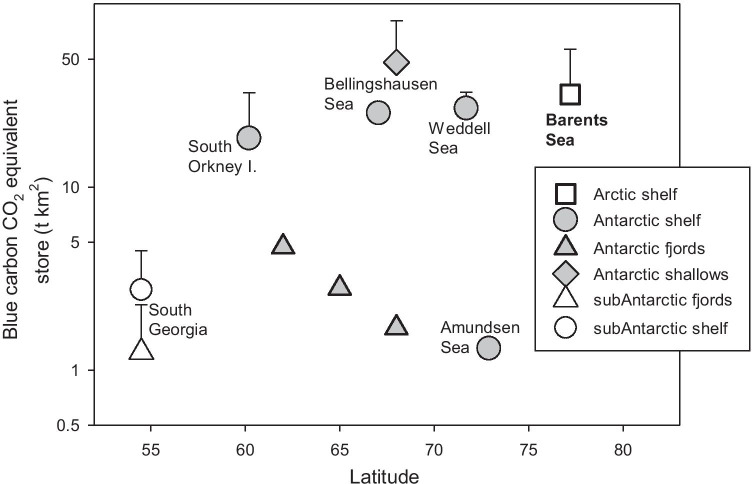
Standing stocks (CO_2_ equivalent) of blue carbon on Arctic and Antarctic continental shelves ( adapted from Souster et al. 2020). Antarctic 5–25 m shallows (diamonds) and fjord (triangles) data are from Barnes (2017) and Barnes et al. (2020)

Nevertheless, initial findings suggest that mean zoobenthic carbon storage (production) varies from ~ 13 t C km^−2^ year^−1^ in coastal shallows reducing to ~ 5 t C km^−2^ year^−1^ within 300-m depth and ~ 1.5 t C km^−2^ year^−1^ below this (Arntz et al. 1994; Brey and Gerdes 1998; Barnes 2017). Across continental shelf depths, and including the carbon in carbonate, this is estimated up to ~ 60 Mt C around the 4.4 million km^2^ Antarctic shelf, including a doubling over the last 25 years coincident with sea ice losses (Barnes et al. 2018). However, where sea ice had increased (e.g. in parts of the Weddell and Ross seas), zoobenthic carbon storage decreased (Pineda Metz et al. 2020). Bryozoa in the Amundsen Sea, for example (whose annual production production/biomass [P/B] ratio is median for Antarctic benthos, see Pinkerton et al. 2010), accumulated about half of the carbon measured in the Scotia Sea, which has only half the annual sea ice duration of the Amundsen Sea (Barnes 2015). Sea ice-seabed carbon relationships via primary production changes are unlikely to be simple. This is because:

- It matters when and where sea ice is lost. Only sea ice losses over the continental shelf have been evaluated and losses during winter (darkness) are unlikely to have as pronounced (light related) influences as those in spring or autumn. In coastal waters, the timing of sea ice loss has been predicted to have potentially profound effects on the growth of macroalgae in areas where it is currently not found. For example, a small increase in ice-free days (~ 10 days) can result in orders of magnitude greater light availability and promote successful macroalgal invasions to new habitats (Clark et al. 2013).
- Responses are location and habitat dependent. As sea ice algae and sea ice habitat are lost, the size and composition of open-water phytoplankton changes, effecting which consumers may benefit or lose out (see Rogers et al. 2020).
- Coastal sea ice losses can increase ice scour in the shallows (because of facilitated iceberg movement, see Fig. 3) and thus benthos mortality leading to reduced net benthic carbon storage in the productive shallows (Barnes 2017). However, Barnes (2017) has shown that this is more than compensated for by greater secondary production in deeper (> 100 m) water driven by longer phytoplankton blooms. This may also vary regionally, as areas where strong katabatic winds dominate, icebergs are blown offshore and scour effects are not as prominent (see Kortsch et al. 2012). This is true for areas in east Antarctica such as the Windmill Islands and the Vestfold Hills, although it has not been quantified to date. Katabatic winds also have the potential to advect locally produced phytoplankton biomass (Lundesgaard et al. 2019), and storms can promote particle aggregation and carbon export to the seabed in some places (e.g. Isla et al. 2009).
- Despite showing similar standing stocks of zoobenthic carbon (Fig. 4), Arctic benthos appears to be responding differently to sea ice losses than around Antarctica, at least in the Barents Sea (see Souster et al. 2020). Phytoplankton blooms are also increasing there (Arrigo and van Dijken 2011) but Souster et al. (2020) only found a relationship between zoobenthic carbon and water flow velocity (i.e. no relationship with sea ice duration). Carbon flux may be more important than the magnitude of overlying productivity in predicting the potential for carbon storage/sequestration from sea ice changes. However, Reigstad et al. (2011) has shown that in the Barents Sea, an increase in productivity could be buffered by processing within the benthic community, which increases storage but not sequestration by burial. Therefore, long-term storage and sequestration, which is maximally important for climate change mitigation, may not occur across all polar regions.
- Changes in sea ice cover do not only affect primary production and blue carbon production directly, but also lead to complex changes in the coupling of ocean and atmosphere, e.g. through altered albedo and modified heat and moisture exchange. For example, with less ice, more heat is absorbed by sea water in summer which could translate to more and/or faster algal growth (Arrigo et al. 2008).

The magnitude of blue carbon gains from sea ice losses (0.06 Gt C year^−1^ or 0.22 Gt CO_2_e a^−1^, see Barnes et al. 2018) make them a small carbon sink, but comparable with lower latitude mangrove swamps and seagrass beds (mitigation potentials of 0.18–0.29 GtCO_2_e a^−1^ estimated for mangroves and 0.22–0.7 GtCO_2_e a^−1^ for seagrasses, see Hoegh-Guldbergh et al. 2019: Pörtner et al. 2021). However, because their polar productivity increases with climate change, unlike low-mid-latitude carbon sinks, this makes blue carbon gains from sea ice losses (Fig. 2a) the largest natural negative (mitigating) feedback on climate change. The previous largest known was the Arctic Taiga forest growth, following closely by Arctic snow and ice retreat (Housset et al. 2015). Whilst sea ice changes alter existing carbon sink dynamics, other marine ice losses, such as ice shelves, generate new ones.

## Ice shelf collapse

Where the huge domed ice sheet that covers most of Antarctica meets the sea, it advances as a floating extension, termed an ice shelf (Fig. 2b). Ice shelves can also extend from glaciers and are often referred to as ice tongues. Unlike the massive seasonal change in sea ice, ice shelves have semi-permanency over 10,000–100,000 years, and within that, parts of them can be more dynamic than others (advancing in some places and collapsing in others). Although there is life under floating ice shelves, it is sparse, often limited in diversity, and thought to be very slow growing (Littlepage and Pearse 1962; Ingels et al. 2021). All studies of life found under ice shelves (major studies reviewed in Ingels et al 2021; Griffiths et al. 2021) indicate that, in general, communities under ice shelves are similar to those found in the deep sea, which are dominated by a limited group of benthic animals that can live in food-poor environments, such as sponges, brittle stars and cold-water corals. Life is thought to be sparse under ice shelves due to the lack of vertical food movement from the upper layers of the ocean to the seafloor, which are blocked by the ice. The small amounts of food feeding sub-ice shelf communities are thought to be either produced locally under ice-shelves or advected in by currents or tides.

So far, due to the difficulties in studying sub-ice shelf communities, we only know of 36 sample sites across the 1.5 million km^2^ Antarctic ice shelves, which are found at eight ice shelves and a marine glacier tongue (Fig. 6). Antarctica has 45 ice shelves, which means that less than 20% of ice shelves currently have any data, and more than half of sampled ice shelves only have one sampling site. More than a third of sampling sites found no visible benthic life (from imagery), which greatly influences our understanding of sparsity under ice shelves. However, several sampling sites from under the Amery and Ross ice shelves have found greater diversity and abundance of life, likely due to proximity to inflowing currents or ice-shelf edge (Bruchhausen et al. 1979; Riddle et al. 2007; Post et al. 2014). It is speculated that each sub ice-shelf community is quite different, more like a deep-sea subset of the greater region that it sits within. However, limitations in sampling, and the use of different sampling methods, make comparisons difficult between sub ice-shelves communities and non-sub ice-shelf communities.

Cape et al. (2014) found that the disintegration of Larsen A and B ice shelves led to the embayment functioning as coastal heat polynyas with net primary productivity rates of ~ 1200 mg C m^−2^ day^−1^ and annual rates reaching ~ 200 g C m^−2^ year^−1^. These phytoplankton blooms in such large coastal embayments can turn the seabed from a dark food-poor desert, into a diverse and dynamic benthic community, under the right conditions, such as those found on the more productive parts of the Southern Ocean seabed, over several decades (Ingels et al. 2021). In addition to making more seabed available for productivity through ice loss, icebergs (from ice shelf break up) release dropstones onto the seabed as they melt. These dropstones are important in the creation of habitats and building seafloor biomass across the Southern Ocean (Zielger et al. 2017; Post et al. 2017). However, it is so far not possible to calculate additional carbon captured from this process, as dropstones can damage seafloor animals, and their impact is likely to vary, depending on where they fall. A decade ago, it was estimated that ice shelf collapse captured an extra ~ 13 Mt CO^2^, equivalent to a new 10,000 ha tropical forest (Peck et al. 2010). Later research into carbon accumulation on the seabed after the Larsen A ice shelf collapsed showed that growth was double what was previously expected (Filinger et al. 2013).

However, neither of these studies had considered the influences, other than making space on the seabed available for colonisation that the giant icebergs breaking away could have on carbon capture and storage. When ice shelf fronts collapse, such as along the northern Antarctic Peninsula, they often create vast tabular icebergs, several hundred metres thick and thousands of square kilometres in area. New phytoplankton blooms were evident in the wake of giant iceberg paths driven by macronutrient release from iceberg melting (Duprat et al. 2016). This can be especially important away from the coast, where iron and other macronutrient limitations stifle phytoplankton blooms (Henley et al. 2020). The fate of this extra primary production is yet to be quantified. Giant icebergs also have the potential to collide with the deep seabed scouring tens to hundreds of kilometres of seabed and destroying much of the benthos established there (Gutt 2001). Many Antarctic giants do not appear to collide with the seabed because they are carried northwards in a clockwise spiral around the Southern Ocean; but those which do mainly hit a limited number of hotspots (due to terrain rises). After consideration of ice scour–induced benthic carbon losses, the net carbon gains of ice shelf collapse and resultant giant iceberg formation is estimated at 4–40 Mt C year^−1^ or ~ 0.1 Gt CO_2_e (based on blooms and benthic carbon accumulation in newly created embayments and iceberg fertilisation along their tracks, Barnes et al. 2018).

To date, we still know very little about under ice shelf communities (Fig. 6) or how much the dynamics of colonisation, after ice shelf collapse, vary between localities, and so all estimates of carbon gains are likely to have considerable associated error. However, our limited knowledge of sub-ice shelf and post ice-shelf collapse communities indicate that even though each ice shelf could be very different in its starting composition, there is a general trend towards increased growth, community complexity, and diversity of life on the seabed, several decades after ice-shelf collapse (reviewed in Ingels et al. 2021). Overall, the carbon sink size resulting from recent ice shelf collapses is globally small (dwarfed by wetlands in the US alone, which store 11.52 Pg C, see Nahlik and Fennessy 2016), despite ice shelves fringing 75% of the vast Antarctic continent. However, the fact that blue carbon gains (like those from sea ice losses) increase as ice shelves recede and collapse means that Antarctic blue carbon is an ecosystem service with societal and economic value worth protecting (estimated at £0.65 and £1.76 billion (~ 2.27 billion USD) for sequestered carbon in the benthos around the continental shelf in Bax et al., 2021). This is the third largest known negative feedback on climate change (Barnes et al. 2018).

## Glacier retreat

Glacier retreat can refer to both reductions in the floating tongue and grounding lines (which are the point at which glaciers are in contract with the ground rather than floating), but the focus here in the current work is on the floating tongue. The nearly 15,000 Antarctic marine glaciers have a global area of 137,866 km^2^, which is small in terms of areal extent and blue carbon. The fjords, along which glacier retreat are common globally in subpolar and polar environments. Although the ~ 240 glaciers terminating along the West-Antarctic Peninsula (WAP) have dominated scientific attention, there are important glaciers and fjords elsewhere around Antarctica, such as in Pine Island Bay, Prydz Bay, Vestfold Hills throughout the Ross Sea coast and elsewhere. This third source of marine ice loss (glacier retreat, Fig. 2c) although small may be disproportionally important to both biodiversity and carbon sequestration because of the nature of fjords created by glacier retreat. Climate change is increasing the proportion of glaciers in retreat (90%) and the rate at which they retreat in Antarctica (Cook et al. 2016). Like ice shelf collapses, glacier retreat creates new, highly productive phytoplankton blooms (and outside the High Antarctic undoubtedly macroalgae forests as well). Rich and abundant benthic communities are known to occur in Antarctica’s opening fjords (Grange and Smith 2013). However, both positive and negative impacts of glacial retreat have been noted along Antarctic fjords, e.g. southern Antarctic Peninsula fjords have little ice melt and lower sedimentation rates compared to those in the northern Antarctic Peninsula, where sedimentation can be high because those glaciers have retreated onto land (see Grange and Smith, 2013; Sahade et al. 2015). Typically, steep fjord sides and muddy floors should provide ideal sequestration possibilities for new production and become important recent low-energy shallow coastal environments. New life, measured as increased biomass of epizoobenthos, emerging in the wake of glacier retreat, were found to now store 12–36 t C year^−1^ in each fjord, so the 216 retreating along the WAP may store 3000–5000 t C year^−1^ (Barnes et al. 2020).

These blue carbon storage gains may be small, but Antarctica gains an important new habitat, which is quite different (in terms of energy and likely water residence time) and could become both biodiversity and blue carbon hotspots over the coming decades, because fjords have some of the highest sequestration potential (estimated at 25% or > 1000 t C year^−1^ in just WAP fjords see Barnes et al. 2020, Smith et al., 2015). To be successfully sequestered, the carbon needs to be removed from the system to prevent recycling to occur. Burial is one of the most efficient ways of removing organic matter from the carbon cycle. Per unit area, fjord systems have been found to have burial rates a hundred times higher than the global ocean average and approximately five times higher than the continental shelf (Smith et al., 2015). Sedimentation rates, which are facilitated by burial, are higher closer to a glacier and decrease with relative distance (Eidam et al. 2019; Sahade et al. 2015). Whilst case studies for individual fjords in Antarctica have shown comparatively small accumulation rates of up to 7.9 mm/year, in situ sedimentation rates on the Antarctic shelf have been found to be a magnitude lower than this (Eidam et al. 2019; Bodungen et al. 1986). Polar fjords have shown greater sediment accumulation rates compared to Northern European, Chilean and New Zealand fjords, with the Arctic leading substantially (Smith et al. 2015). Thus, the retreat of polar glaciers and consequential increase in carbon sequestration is an important instrument in climate mitigation.

A potential trade-off between blue carbon gains and losses in coastal waters is in fjords that are currently covered by sea ice for most of the year (for example many around East Antarctica and the southern Bellingshausen Sea). Here, a decrease in ice duration could lead to the establishment of macroalgal-dominated communities (where there are currently none) (for Arctic examples see Kortsch et al. 2012) and the loss of invertebrate-dominated communities that occur under ice, which may be outcompeted by macroalgae (Clark et al. 2013). As macroalgae grows faster and more seasonally, its annual growth and biomass may be greater than that of the invertebrate dominated communities, but this has not been tested. An important area for new research is investigating how deep basins of fjords, away from the influence of sunlight, are altered by ice loss, warming and other climate change stressors.

Growth and production estimates from fjords do not match those of typical Antarctic shelf (see Arntz et al 1994). Establishment, survival and growth of benthos along newly emerging fjords may initially be restricted by freshening and high sedimentation loads (Sahade et al. 2015). As well as calved iceberg scour potential, there is another source of carbon loss associated with glacier retreat. Glacier ice contains 0.02–0.04 mg carbon L^−1^ which is equivalent to 18.3–36.7 t km^3^ (Legrand et al. 2013). The fate of this carbon discharged with calved icebergs is unknown but this offsets about 0.3–1% of blue carbon gains from increased seabed benthic communities. The net equivalent blue carbon gains from glacier retreat along Antarctic fjords are moderate and comparable to ~ 140 ha of tropical forest, but more important owing to their much higher sequestration potential. The error associated with polar fjord blue carbon gains, as with sea ice and ice shelves, is considerable (Barnes et al. [2020] found estimated values for three fjords to be 50% of the same fjords measured). This is due to their being greater variability in glacier retreat rates between fjords (Cook et al. 2016), and with the few fjords that have been investigated and those that have, they have shown that biomass differs by more than a factor of 3 (Grange and Smith 2013; Sahade et al. 2015; Barnes et al. 2020).

## Potential polar blue carbon gains from other influences

Mregions of polar coasts that have increasing annual periods of ice-free conditions or are becoming entirely ice-free in response to ocean warming Until recently, little consideration ha d been given to kelp and pseudo-kelp (macroalgal) forests primarily because sequestration does not occur in situ and is more difficult to measure. macroalgal spread storage Ocean acidification is occurring more rapidly in polar waters than in other regions, because cold water absorbs and holds more carbon dioxide, increasing acidity (McNeil and Matear 2008; Fabry et al. 2009). Ocean acidification is predicted to increase the rate of photosynthesis and growth of many non-calcifying marine macroalgae under elevated CO_2_ (Koch et al. 2013; Young and Gobler 2016). Although a decrease in pH may also alter macroalgal communities significantly, some species are more tolerant of long-term increases in CO_2_ levels (dissolved in the ocean) than others (Porzia et al. 2011). More research is needed on the effects of ocean acidification on polar macroalgae, but it could lead to further gains in blue carbon capture, storage and sequestration if biomass gain by macroalgae is not offset by loss and faster dissolution of calcifying organisms (Agostini et al., 2018). Figuerola et al. (2021) have shown, in a recent meta-analysis of vulnerability of Southern Ocean species to ocean acidification, that mineralogical composition was the most important characteristic, with those containing calcitic, aragonitic and high Magnesium calcite skeletons which were at much greater risk than those of low-magnesium calcite.

## Future risk, threats and actions needed

**Fig. 5:**
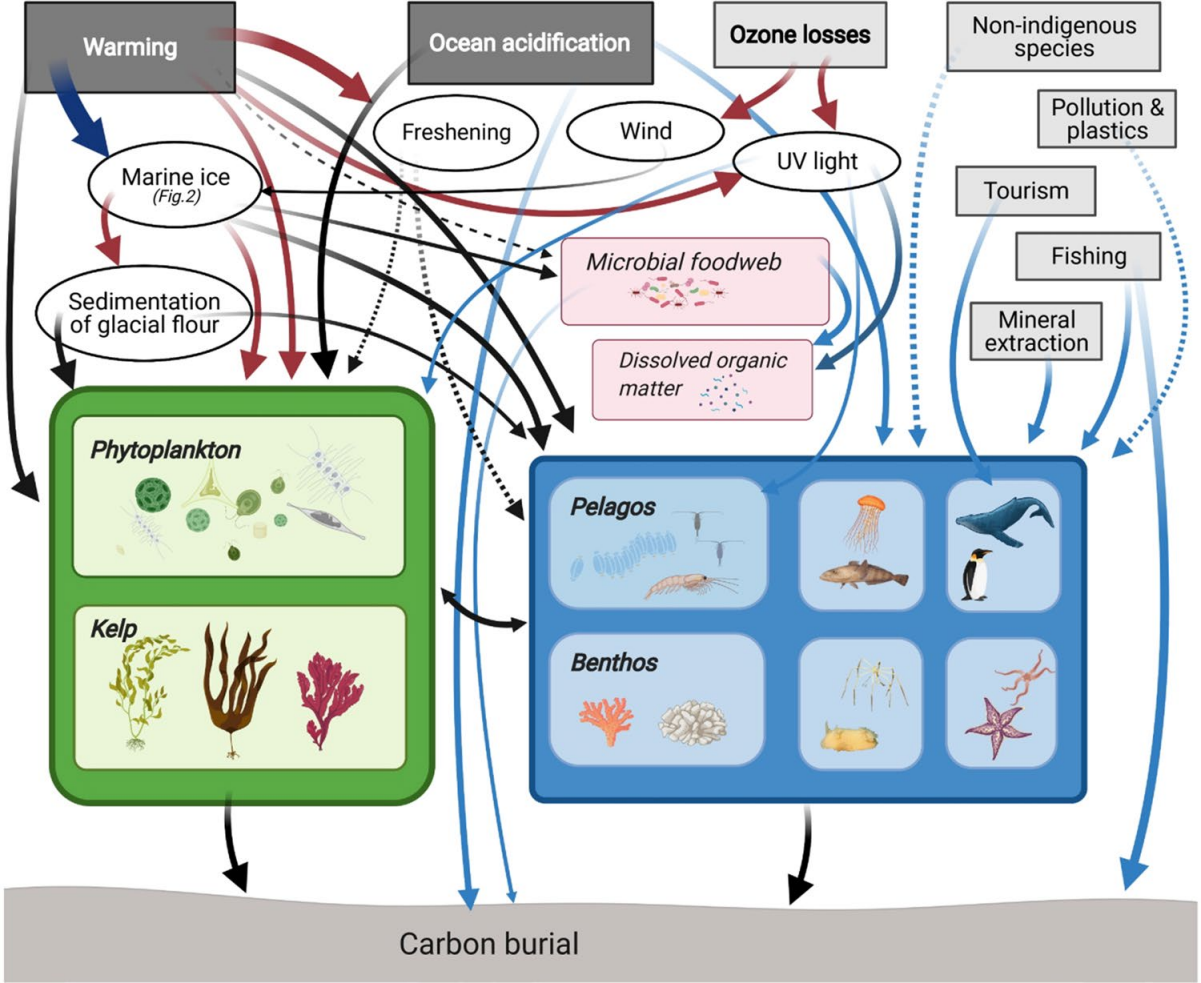
Schematic showing stressors and their links to Antarctic blue carbon capture, storage and sequestration. The boxes are primary effects of climate change (dark grey rectangles), secondary effects of climate change (ovals), non-climate change stressors (grey rectangles), primary producer (= carbon capture; green rectangle), microbial and dissolved organic matter (= microbial loop; red rectangles) and secondary producers (= carbon storage; blue rectangle). The arrows are decreases (blue), changes (black) and increases (red). Dashed lines are potential influences, as yet unquantified. More detail is these stressors are given in Gutt et al (2015), Rogers et al. (2020) and Morley et al. (2020)

There are many and complex threats to polar food webs and ecosystem functioning (Fig. 5) which are the basis of polar blue carbon (Gutt et al. 2015). A recent Marine Ecosystem Assessment of the Southern Ocean (MEASO) specifically considers the future of ecosystem services, including biological carbon sequestration (Cavanagh et al. 2021) and the stressors on these (Morley et al. 2020). Because the Southern Ocean and Antarctic shelves are unusually deep, negative biological responses to many of these stressors may take longer than elsewhere to be detected (because of both accessibility and lack of baselines); however, substantial biological change has already been observed (Trivelpiece et al. 2011; Rogers et al. 2020). These include complex interactions between stressors (Gutt et al. 2015; Brasier et al. 2021). Such literature also suggests that the strongest threats come in the form of catastrophic ecosystem disruption by combined elements of climate change: ocean acidification, freshening, sedimentation and other physical changes, such as ocean warming exacerbating ice-shelf basal melting through tidal changes (Brasier et al. 2021; Mueller et al. 2018). Projected ocean acidification would seem to pose major problems, not just to the cost of growth and skeletons, but also to perseverance of carbonaceous material on the seabed after death (Figuerola et al., 2021). Some anthropogenic stressors are both directly and indirectly influenced. For example, non-indigenous species (NIS) invasions, which are facilitated by aspects of climate change, could also prove highly and permanently disruptive to benthic biodiversity (Hughes et al. 2020). As with NIS, plastic pollution is also starting to reach the polar regions and polar food webs, but to date the impact of this remains unclear (Waller et al. 2017; Rogers et al. 2020). The Antarctic ozone hole is one of the largest and deepest. Harmful UV radiation is costly for near-surface phyto- and zooplankton as these organisms must produce UV protection (e.g. pigments and repair systems, see Rogers et al. 2020). UV radiation also causes photochemical degradation of dissolved organic matter to CO_2_, and thus forms an important sink of pelagic carbon in addition to bacterial degradation (Mopper et al. 1991). It is unclear whether the bacterial degradation of pelagic organic matter will increase with rising temperatures; however, bacteria will likely respond positively to a decrease in sea ice (Kirchman et al. 2009), and to ocean acidification (Hancock et al. 2020). An increase in bacterial and photochemical degradation of pelagic organic matter results in a decreased export to the sediments (Fig. 5). All the discussed stressors can be decreased but require coordinated and societal action globally. In contrast, one stressor can be targeted regionally, and it has proved devastating almost everywhere around the planet: harvesting of marine resources, typically non-selective fishing practises. Seabeds targeted by demersal fishing can be rich in habitat engineers and immobilised carbon (‘marine animal forests’, see Rossi and Rizzo 2020) with considerable impacts to carbon-rich sponges, corals and associated fauna (Montseny et al. 2019). The implications of benthic bycatch are more devastating in polar waters. Most polar life develops and grows very slowly (i.e. non endotherms), perhaps an order of magnitude slower than that in warm water (see Arntz et al. 1994). Recovery of ecosystems from bottom trawling may take decades to centuries (Thrush and Dayton 2002) although the pace of benthic recolonization and growth after ice shelf breakout was found to be surprisingly fast in small areas at least (Filinger et al. 2013, Zwerschke et al. 2021).

## Conclusions

**Fig. 6:**
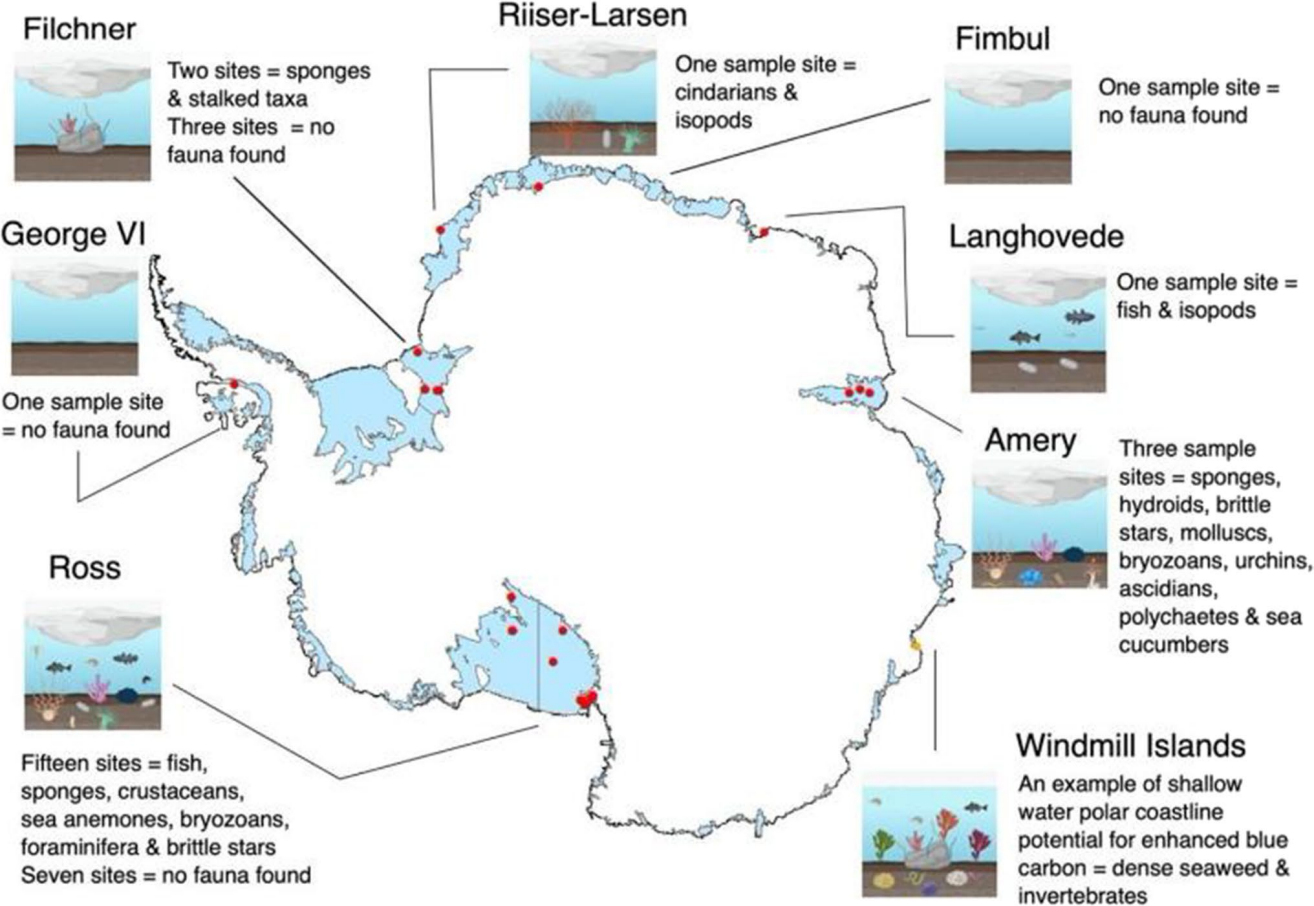
Map of the extensive Antarctic ice-shelves and our current knowledge of life in these sub-ice environments. Sub-ice shelf organisms tend to be from few phyla, and are patchily distributed; therefore, blue carbon gains from the colonisation of both mobile and sessile fauna post-collapse could be substantial

**Fig. 7:**
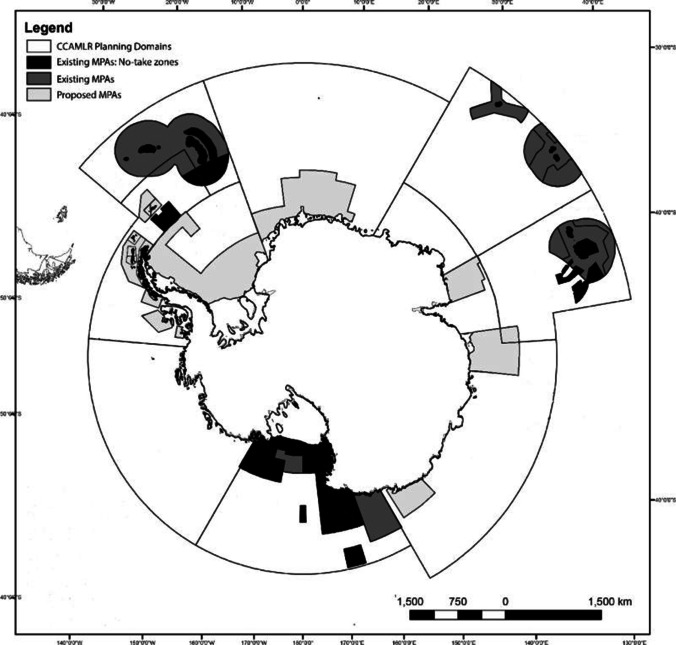
Map of Antarctica showing existing and proposed marine protected areas (MPAs) within the Committee for the Conservation of Marine Living Resources (CCAMLR) domains, adapted from Brooks et al. (2020)

Antarctic continental shelves and coastlines are on the frontline of physical responses to climate change, and such physical changes have proved highly complex in space and time. Unsurprisingly, their interaction with biota and ecosystem services, such as blue carbon feedbacks on climate, is also complex and dynamic (Gutt et al. 2015; Rogers et al. 2020; Morley et al. 2020, and Fig. 5). The work so far has highlighted the importance of all three types of ice loss to blue carbon in polar environments. Yet it has become clear that we are still unaware of the poles’ full potential when it comes to carbon sequestration. One problem is that measuring sedimentation, which essentially drives carbon sequestration, has not been reliably achieved throughout the Antarctic continental seabeds. Furthermore, it is unclear how much of the stored blue carbon will eventually be recycled and how much will be sequestered. It is also noteworthy that the majority of studies estimating blue carbon in polar regions have based their predictions on the expected increase of epifaunal marcrozoobenthos (Barnes et al. 2020) thereby neglecting to include further potential growth for blue carbon posed by infauna colonising soft sediments, organic matter sedimentation and potential increase in benthic bacteria (Barnes 2015; Smith et al. 2015; Souster et al. 2020). Thus, estimated standing stocks of blue carbon in polar waters may appear moderate by global standards but these are likely to be a great underestimation of actual values, and are bound to become even more important as they increase with climate change (a negative [mitigating] feedback loop). This feedback is driven by blue carbon capture to storage relationships with (three forms of) marine ice (Fig. 2). In the shallows, the relationship between blue carbon and marine ice losses is further complicated by (a) blue carbon losses caused by increased iceberg scour (Fig. 3), (b) some growth (blue carbon capture and storage) increases with moderate warming (Ashton et al. 2017), (c) ocean–atmosphere interchange (such as albedo) changes of marine ice loss driving a positive feedback on climate change, and (d) the non-linear nature of seasonal light cycles in relation to timing of annual sea ice loss (Clark et al. 2013). Increasingly, it is being realised that nature-based solutions, such as protection and restoration from direct anthropogenic stressors, have a very important role to play in limiting climate change (Bax et al. 2021; Cavanagh et al. 2021). We suggest that this is especially the case in the polar regions because of these powerful blue carbon negative feedbacks on climate change, and that many of these emerging carbon sinks do not need expensive restoration but more cost-effective simple protection, such as the marine protected areas around South Georgia, the southern South Orkney islands and most recently part of the Ross Sea (Fig. 7). A better understanding of the extent of shallow ecosystems and the role they play in blue carbon capture is needed for most regions of Antarctica, particularly beyond the Peninsula, for example in east Antarctica, where the only studied fjords are in the Vestfold Hills, although more are known to occur in other areas. Isolated coastal ice-free outcrops in Antarctica are usually associated with extensive shallow water ecosystems (for example the Windmill Islands) where macroalgae- or invertebrate-dominated communities contribute to blue carbon capture and sequestration (Fig. 6). How such areas will respond to environmental change is not well understood. Finally, we acknowledge that nowhere around Antarctica has a whole blue carbon budget been calculated. When this is managed (including all elements e.g., Figs. 1–5), we predict that it will show that stocks may have been considerably underestimated.
